# Combined melatonin-adipose derived mesenchymal stem cells therapy effectively protected the testis from testicular torsion-induced ischemia-reperfusion injury

**DOI:** 10.1186/s13287-021-02439-x

**Published:** 2021-06-29

**Authors:** Yen-Ta Chen, Fei-Chi Chuang, Chih-Chao Yang, John Y. Chiang, Pei-Hsun Sung, Yi-Ching Chu, Chi-Ruei Huang, Kuan-Hui Huang, Hon-Kan Yip

**Affiliations:** 1 Division of Urology, Department of Surgery, Kaohsiung Chang Gung Memorial Hospital, Chang Gung University College of Medicine, Kaohsiung, Taiwan; 2 Center for Shockwave Medicine and Tissue Engineering, Kaohsiung Chang Gung Memorial Hospital, Kaohsiung, Taiwan; 3 Department of Obstetrics and Gynecology, Kaohsiung Chang Gung Memorial Hospital, Kaohsiung, Taiwan; 4 Division of Nephrology, Department of Internal Medicine, Kaohsiung Chang Gung Memorial Hospital, Chang Gung University College of Medicine, Kaohsiung, Taiwan; 5 Department of Computer Science and Engineering, National Sun Yat-Sen University, Kaohsiung, Taiwan; 6 Department of Healthcare Administration and Medical Informatics, Kaohsiung Medical University, Kaohsiung, Taiwan; 7 Division of Cardiology, Department of Internal Medicine, Kaohsiung Chang Gung Memorial Hospital, Chang Gung University College of Medicine, Kaohsiung, Taiwan; 8 Institute for Translational Research in Biomedicine, Kaohsiung Chang Gung Memorial Hospital, Kaohsiung, Taiwan; 9 Department of Nursing, Asia University, Taichung, Taiwan; 10 Department of Medical Research, China Medical University Hospital, China Medical University, Taichung, Taiwan; 11 Division of Cardiology, Department of Internal Medicine, Xiamen Chang Gung Hospital, Xiamen, Fujian China

**Keywords:** Testis, Ischemia-reperfusion injury, Melatonin, Adipose-derived mesenchymal stem cells

## Abstract

**Background:**

This study tested the hypothesis that combined melatonin (Mel) and adipose-derived mesenchymal stem cells (ADMSCs) treatment was superior to either one alone on protecting the testis against acute testicular torsion-induced ischemia-reperfusion (TTIR) injury.

**Methods and results:**

Male adult SD rats (n = 30) were equally categorized into group 1 (sham-operated control), group 2 [TTIR/by torsion of right/left testis (i.e., ischemia) with rotated 720° counterclockwise for 2 h, then detorsion (i.e., reperfusion) to the original position for 72 h], group 3 (TTIR + Mel/intraperitoneal administration/50 mg/kg at 30 min after ischemia, followed by 20 mg at 3 h and days 1/2/3 after TTIR), group 4 (TTIR + ADMSCs/1.2 × 10^6^ cells/by tail-vein administration at 30 min after ischemia, followed by days 1/2 TTIR), and group 5 (TTIR + Mel + ADMSCs/tail-vein administration). The result showed that the protein expressions of oxidative-stress (NOX-1/NOX-2/oxidized-protein), apoptotic/mitochondrial-damaged (mitochondrial-Bax/cleaved-caspase3/cleaved-PARP/cytosolic-cytochrome C), and fibrotic (TGF-ß/Smad3) biomarkers as well as testicular damage scores were lowest in group 1, highest in group 2, and significantly higher in groups 3/4 than in group 5, but they showed no difference between groups 3/4, whereas the protein expressions of androgen receptor (AR) and vimentin showed an opposite pattern of oxidative stress (all p < 0.0001). The cellular levels of inflammation (MMP-9/MPO/CD68) exhibited an identical pattern, whereas the numbers of Sertoli cells, α-tubulin, AR and vimentin as well as thickness of seminiferous tubule exhibited an opposite pattern of oxidative stress among the groups (all p < 0.0001).

**Conclusion:**

Mel-ADMSCs effectively protected the testis against TTIR injury.

## Introduction

Testicular torsion due to twisting of the spermatic cord is a serious urological emergency that is most commonly encountered in those of newborns, children, and adolescent males [1, 2]. Testicular torsion causes testicular injury, leading to potentially serious sequelae of infertility and subfertility; thus, rapid diagnosis and prompt treatment are extremely important [3–5]. Undoubtedly, the duration and degree of cord twisting are the two major contributors that are closely related to the severity of the testicular injury [2, 6].

It is well known that testicular ischemia-reperfusion (IR) injury represents the essential pathophysiology of testicular torsion, with ischemia caused by twisting of the spermatic cord, and reperfusion on its subsequent release. Accordingly, the successful treatment of testicular torsion is clearly identified in two distinct steps. First, to quickly release the torsion (i.e., detorsion) by surgical intervention [1–7]. This procedure which is a standard method for treatment of testicular torsion usually poses no difficulty for an interventional urologist. Second, to prevent reperfusion injury by pharmacomodulation. However, currently, no effective pharmaceutics for reducing the gonad damage from reperfusion injury due to the central pathological mechanisms of testicular injury following testicular torsion are not entirely understood, despite the overproduction of reactive oxygen species (ROS)/free radicals and reactive nitrogen species generated during the IR process have been implicated as two of the main factors in cellular and tissue damage [8–10]. Of particular interest is that while the ipsilateral testicular damage after spermatic cord torsion is better clarified, the status of the contralateral testis remains obscure [11–16].

Vast research have revealed that melatonin, a powerful free-radical scavenger and metal chelator [17] with the ability of attenuating oxidative stress [18–20] and inflammatory reactions [21, 22] as well as stabilizing cellular membranes [23], furnished protective effects against sepsis-caused kidney organ damage [24]. Additionally, rich data has demonstrated that stem cell therapies were effective and promising for many disease entities which are refractory to traditional treatment [25–28]. Of these, adipose-derived mesenchymal stem cells (ADMSCs) have been proved to augment angiogenesis and tissue regeneration, leading to improved ischemia-related organ dysfunction, as well as has anti-inflammatory and immunomodulatory capacity for ameliorating IR-induced organ dysfunction [24, 29–31].

Intriguingly, abundant experimental studies have brought to light that IR-caused organ dysfunction and tissue damage are mainly through inflammation, generation of oxidative stress/ROS, and DNA damage which are significantly inhibited by Mel or ADMSCs therapy [21, 22, 24, 27, 29–31]. However, whether cell therapy would offer benefit for protecting the testis from testicular torsion-induced IR (TTIR) injury is currently unknown. Accordingly, this study tested whether combined Mel-ADMSCs therapy would be superior to either one alone for protecting the testis against reperfusion injury.

## Materials and methods

### Ethics

All animal procedures were approved by the Institute of Animal Care and Use Committee at Kaohsiung Chang Gung Memorial Hospital (Affidavit of Approval of Animal Use Protocol No. 2017012401) and performed in accordance with the Guide for the Care and Use of Laboratory Animals.

Animals were housed in an Association for Assessment and Accreditation of Laboratory Animal Care International (AAALAC; Frederick, MD, USA)-approved animal facility in our hospital with controlled temperature and light cycles (24 °C and 12/12 light cycle).

### Isolation of ADMSCs 

Adipose tissue surrounding the epididymis was carefully dissected, excised, and prepared based on our recent reports [24, 32]. In details, after isolation of the adipose tissues, 200–300 μL of sterile saline was added to every 0.5 g of adipose tissue to prevent dehydration. The tissue was cut into < 1 mm^3^ size pieces using a pair of sharp, sterile surgical scissors. Sterile saline (37 °C) was added to the homogenized adipose tissue in a ratio of 3:1 (saline to adipose tissue), followed by the addition of stock collagenase solution to a final concentration of 0.5 units/mL. The centrifuge tubes with the contents were placed and secured on a Thermaline shaker and incubated with constant agitation for 60 ± 15 min at 37 °C. After 40 min of incubation, the content was triturated with a 25-mL pipette for 2–3 min. The cells obtained are placed back to the rocker for incubation. The contents of the flask are transferred to 50-mL tubes after digestion, followed by centrifugation at 600*g* for 5 min at room temperature. The fatty layer and saline supernatant from the tube were poured out gently in one smooth motion or removed using vacuum suction. The cell pellet thus obtained was resuspended in 40 mL saline and then centrifuged again at 600*g* for 5 min at room temperature. After being resuspended again in 5 mL saline, the cell suspension was filtered through a 100-μm filter into a 50-mL conical tube to which 2 mL of saline was added to rinse the remaining cells through the filter. The flow-through was pipetted into a new 50-mL conical tube through a 40-μm filter. The tubes were centrifuged for a third time at 600*g* for 5 min at room temperature. The cells were resuspended in saline. An aliquot of cell suspension was then removed for cell culture in Dulbecco’s modified Eagle’s medium (DMEM)-low glucose medium containing 10% FBS for 14 days. Approximately 2.0–3.0 × 10^6^ ADMSCs were obtained from each rat. Flow cytometric analysis was performed for identification of cellular characteristics after cell labeling with appropriate antibodies on day 14 prior to transfusion.

### The procedure and protocol of testicular torsion to induce acute TTIR injury

The procedure and protocol were based on the previous report with some modifications [33]. In detail, pathogen-free, adult male Sprague-Dawley (SD) rats weighing 320–350 g (Charles River Technology, BioLASCO Taiwan Co. Ltd., Taiwan) were used in the present study. All animals were anesthetized by inhalational 2.0% isoflurane, placed in a supine position on a warming pad at 37 °C for midline laparotomies. The sham-operated control (SC) group received ilioinguinal incisions only, while IR was induced in other groups of animals by rotating 720° in a counterclockwise direction and maintained for 2 h by fixing the right and left testes to the scrotum with a 4–0 silk suture. Detorsion (i.e., returned to the normal position) was then performed by untwisting the testes to original position and maintaining the position.

The animals (n = 30) were equally categorized into group 1 (i.e., SC, only by a sham operation of the left and right testes without any IR procedure), group 2 [TTIR, i.e., by torsion of right and left testes (i.e., ischemia) with 720° counterclockwise rotation for 2 h, then detorsion (i.e., reperfusion) to the original position for 72 h], group 3 [TTIR + Mel (intraperitoneal administration of Mel 50 mg/kg at 30 min after ischemia, followed by 20 mg at 3 h and days 1, 2, and 3 after IR injury)], group 4 [TTIR + ADMSCs (1.2 × 10^6^ cells by tail intravenous administration at 30 min after ischemia, followed by days 1 and 2 after IR injury)], and group 5 (TTIR + Mel + ADMSCs)], respectively. The dosages of Mel and ADMSCs utilized in the present study were based on our previous studies [24, 27, 29, 30].

### Histopathological finding of examination of testicular specimen after IR procedure

Testes were dissected from the abdomen, immersed in 10% formalin for 24 h, and embedded in paraffin. First, each testis was morphologically evaluated. Additionally, the tissue sections of 5 μm were sliced and stained with hematoxylin and eosin (i.e., H&E). The specimens of H&E stain were carefully examined by light microscopy.

### Immunohistochemical (IHC) and immunofluorescent (IF) staining

The procedures of IF and IHC staining were described in detail in our previous reports [24, 27, 29, 30]. For IHC and IF staining, rehydrated paraffin sections were first treated with 3% H_2_O_2_ for 30 min and incubated with Immuno-Block reagent (BioSB) for 30 min at room temperature. Sections were then incubated with primary antibodies specifically against matrix metalloproteinase (MMP)-9 (1:200, Thermo Fisher), α-tubulin (1:500, Santa Cruz), myeloperoxidase (MPO) (1:200, Bio SB), androgen receptor (AR) (1:100, Santa Cruz), vimentin (1:500, Santa Cruz), and CD68 (1:200, Abcam), while sections incubated with the use of irrelevant antibodies served as controls. Three sections of the testicular specimen from each rat were analyzed. For quantification, three randomly selected HPFs (× 200 or × 400 for IHC and IF studies) were analyzed in each section. The mean number of positively stained cells per HPF for each animal was determined by summation of all numbers divided by 9.

### Western blot analysis

The procedure and protocol for Western blot analysis were based on our previous reports [24, 27, 29, 30]. Briefly, equal amounts (50 μg) of protein extracts were loaded and separated by SDS-PAGE using acrylamide gradients. After electrophoresis, the separated proteins were then transferred electrophoretically to a polyvinylidene difluoride (PVDF) membrane (Amersham Biosciences). Nonspecific sites were blocked by incubation of the membrane in blocking buffer [5% nonfat dry milk in T-TBS (TBS containing 0.05% Tween 20)] overnight. The membranes were then incubated with the indicated primary antibodies against cleaves caspase 3 (1:1000, Cell Signaling), cleaved poly(ADP-ribose) polymerase (c-PARP) (1:1000, Cell Signaling), NOX-1 (1:750, Sigma), NOX-2 (1:1000, Sigma), phosphorylated (p)-Smad3 (1:1000, Cell Signaling), transforming growth factor (TGF)-ß (1:1000, Abcam), mitochondrial Bax (1:1000, Abcam), cytosolic cytochrome C (1:1000, BD), mitochondrial cytochrome C (1:1000, BD), androgen receptor (AR) (1:1000, Abcam), and vimentin (1:1000, Abcam) for 1 h at room temperature. Horseradish peroxidase-conjugated anti-rabbit immunoglobulin IgG (1:2000, Cell Signaling) was used as a secondary antibody for 1-h incubation at room temperature. The washing procedure was repeated eight times within 1 h. Immunoreactive bands were visualized by enhanced chemiluminescence (ECL; Amersham Biosciences) and exposed to Biomax L film (Kodak). For the purpose of quantification, ECL signals were digitized using Labwork software (UVP).

### Assessment of oxidative stress

The procedure for assessing the protein expression of oxidative stress has been described in detail in our previous reports [24, 27, 29, 30]. The Oxyblot Oxidized Protein Detection Kit was purchased from Chemicon (S7150). DNPH derivatization was carried out on 6 μg of protein for 15 min according to the manufacturer’s instructions. One-dimensional electrophoresis was carried out on 12% SDS/polyacrylamide gel after DNPH derivatization. Proteins were transferred to nitrocellulose membranes which were then incubated in the primary antibody solution (anti-DNP 1: 150) for 2 h, followed by incubation in the secondary antibody solution (1:300) for 1 h at room temperature. The washing procedure was repeated eight times within 40 min. Immunoreactive bands were visualized by enhanced chemiluminescence (ECL; Amersham Biosciences) which were then exposed to Biomax L film (Kodak). For quantification, ECL signals were digitized using Labwork software (UVP). For oxyblot protein analysis, a standard control was loaded on each gel.

### Histological measurement of testicular injury and criteria for seminiferous tubules injury score

The testicular specimens were sectioned at 5 μm for light microscopy of H&E stain for each animal. Three testicular sections from each rat were analyzed and three randomly selected high-power fields (HPFs; × 200) were examined in each section. The morphologic destruction score of seminiferous tubules was evaluated in a blinded fashion. The following scoring system was adopted: 0 = no detectable morphologic destruction; 1 = < 15% of morphologic destruction; 2 = 15–25% of morphologic destruction; 3 = 26–50% of morphologic destruction; 4 = 51–75% of morphologic destruction; and 5 = 76–100% of morphologic destruction/HPF. The procedure and protocol of scoring has been described by our previously reported [34].

### Statistical analysis

Quantitative data were expressed as mean ± SD. Statistical analysis was adequately performed by ANOVA followed by Bonferroni multiple-comparison post hoc test. SAS statistical software for Windows version 8.2 (SAS institute, Cary, NC, USA) was utilized for the analysis. A probability value < 0.05 was considered as statistical significance.

## Results

### The protein expressions of apoptotic and oxidative-stress biomarkers by 72 h after IR procedure (Fig. 1)

To determine whether the apoptosis and oxidative stress signalings were upregulated after IR procedure, the Western blot analysis was performed. The result showed that the protein expressions of mitochondrial Bax, cleaved caspase 3, and cleaved PARP, three indices of apoptosis, were lowest in group 1 (i.e., SC), highest in group 2 (TTIR only), and significantly lower in group 5 (TTIR + Mel + ADMSCs) than in groups 3 (TTIR + Mel) and 4 (TTIR + ADMSCs), but they exhibited no difference between groups 3 and 4. Consistently, the protein expressions of NOX-1, NOX-2, and oxidized protein, three indicators of oxidative stress, displayed an identical pattern of apoptosis.

**Fig. 1:**
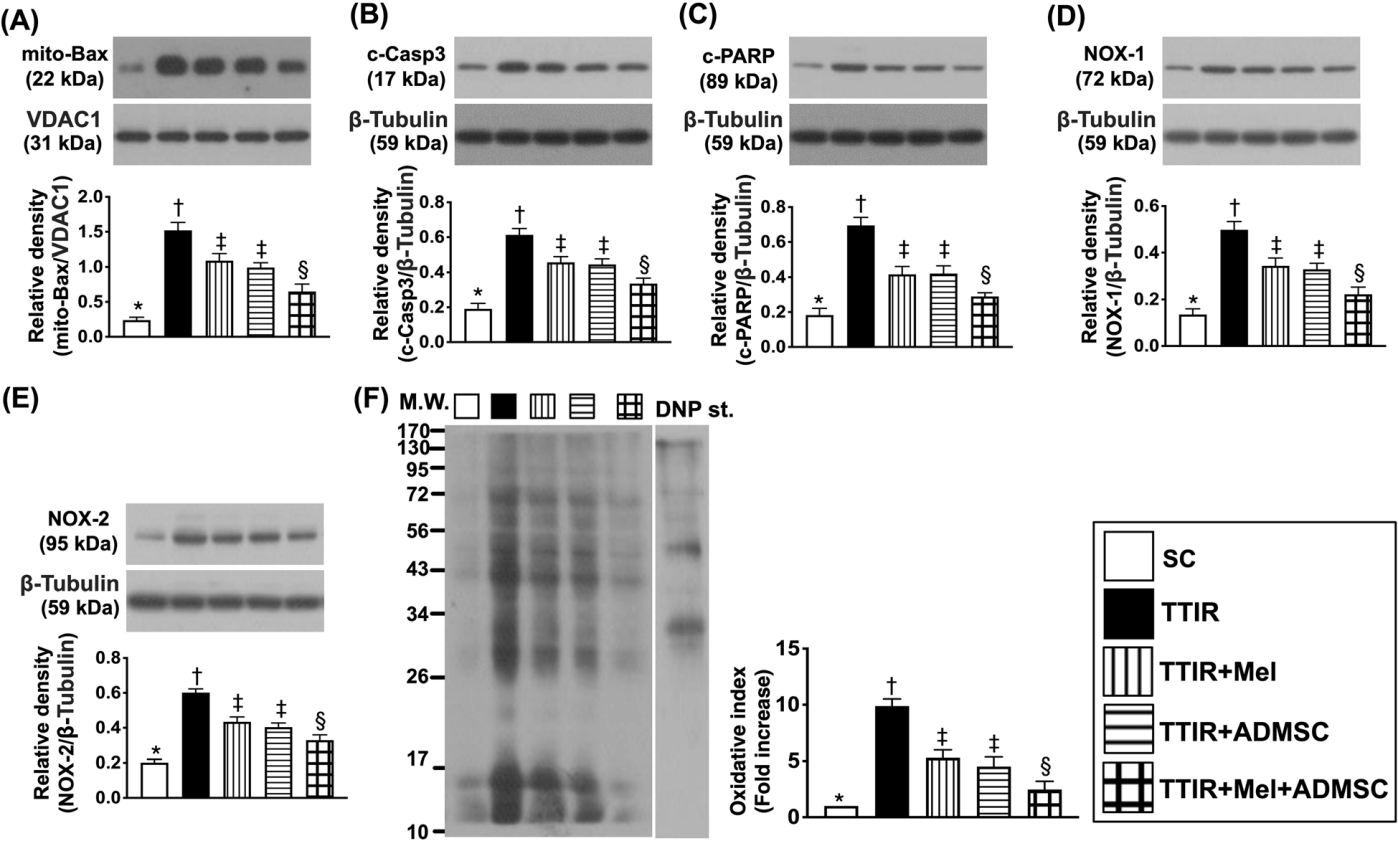
Mel-ADMSCs therapy suppressed the protein expressions of apoptosis and oxidative stress by 72 h after TTIR procedure. **A** Protein expressions of mitochondrial (mito)-Bax, * vs. other groups with different symbols (†, ‡, §), p < 0.0001. **B** Protein expression of cleaved caspase 3 (c-Casp3), * vs. other groups with different symbols (†, ‡, §), p < 0.0001. **C** Protein expression of and cleaved poly (ADP-ribose) polymerase (c-PARP), * vs. other groups with different symbols (†, ‡, §), p < 0.0001. **D** Protein expression of NOX-1, * vs. other groups with different symbols (†, ‡, §), p < 0.0001. **E** Protein expression of NOX-2, * vs. other groups with different symbols (†, ‡, §), p < 0.0001. **F** Oxidized protein expression, * vs. other groups with different symbols (†, ‡, §), p < 0.0001 (note: left and right lanes shown on the upper panel represent protein molecular weight marker and control oxidized molecular protein standard, respectively). M.W., molecular weight; DNP, 1-3 dinitrophenylhydrazone. All statistical analyses were performed by one-way ANOVA, followed by Bonferroni multiple comparison post hoc test (n = 6 for each group). Symbols (*, †, ‡, §) indicate significance (at 0.05 level)

### Protein expressions of fibrotic and mitochondrial-damaged markers by 72 h after IR procedure (Fig. 2)

To elucidate the therapeutic impact of Mel-ADMCSs on regulating the fibrosis and DNA-damaged signalings, the Western blot was utilized. The result showed that the protein expressions of Smad3 and TGF-ß, two indicators of fibrosis, were lowest in group 1, highest in group 2, and significantly lower in group 5 than in groups 3 and 4, but they showed no difference between the latter two groups. Additionally, the protein expression of cytosolic cytochrome C, a mitochondria damage biomarker, exhibited a similar pattern whereas the protein expression of mitochondrial cytochrome C, an indicator of mitochondrial integrity, exhibited an opposite pattern of fibrosis among the five groups.

**Fig. 2:**
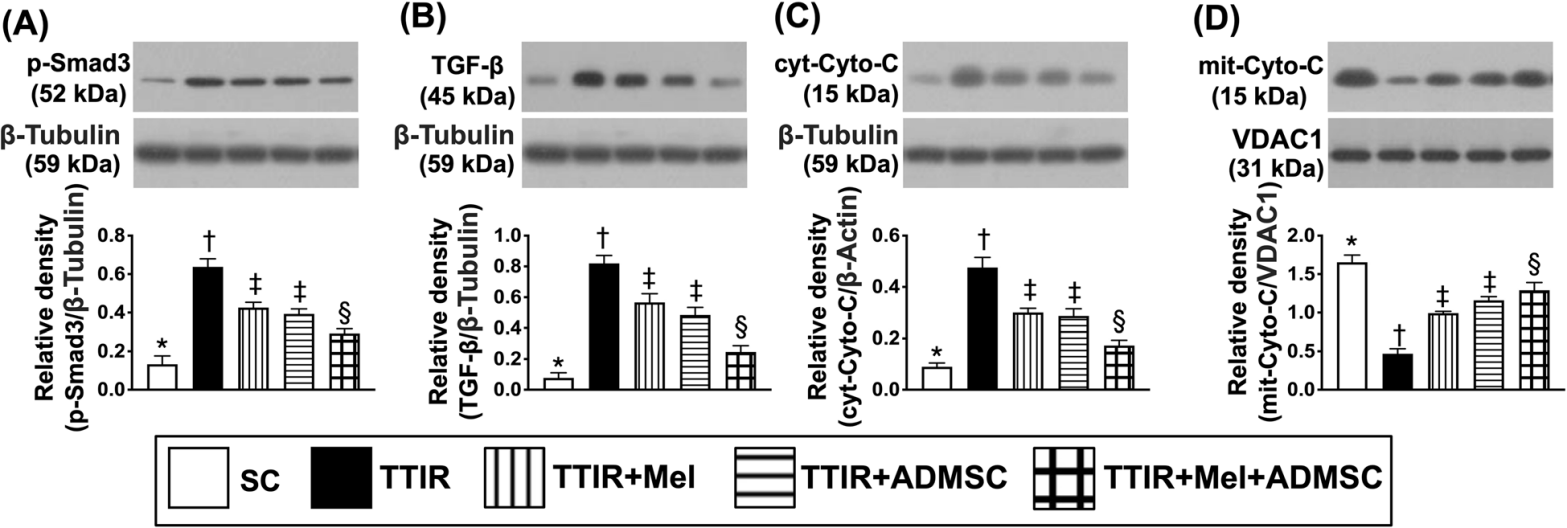
Mel-ADMSCs therapy inhibited the protein expressions of fibrosis and mitochondrial-damaged biomarker by 72 h after TTIR procedure. **A** Protein expression of Smad3, * vs. other groups with different symbols (†, ‡, §), p < 0.0001. **B** Protein expression of transforming growth factor (TGF)-ß, * vs. other groups with different symbols (†, ‡, §), p < 0.0001. **C** Protein expression of cytosolic cytochrome C (cyt-Cyto-C), * vs. other groups with different symbols (†, ‡, §), p < 0.0001. **D** Protein expression of mitochondrial cytochrome C (mit-Cyto-C), * vs. other groups with different symbols (†, ‡, §), p < 0.0001. All statistical analyses were performed by one-way ANOVA, followed by Bonferroni multiple comparison post hoc test (n = 6 for each group). Symbols (*, †, ‡, §) indicate significance (at 0.05 level)

### Protein and cellular expressions of androgen receptor and vimentin by 72 h after IR procedure (Fig. 3)

We further evaluated the expressions of vimentin and AR, two indicators of integrity of testis and its apparatus, with Western blot and microscope. The result showed that the protein and cellular expressions of vimentin and AR were highest in group 1, lowest in group 2, and significantly higher in group 5 than in groups 3 and 4, but they were similar in groups 3 and 4.

**Fig. 3:**
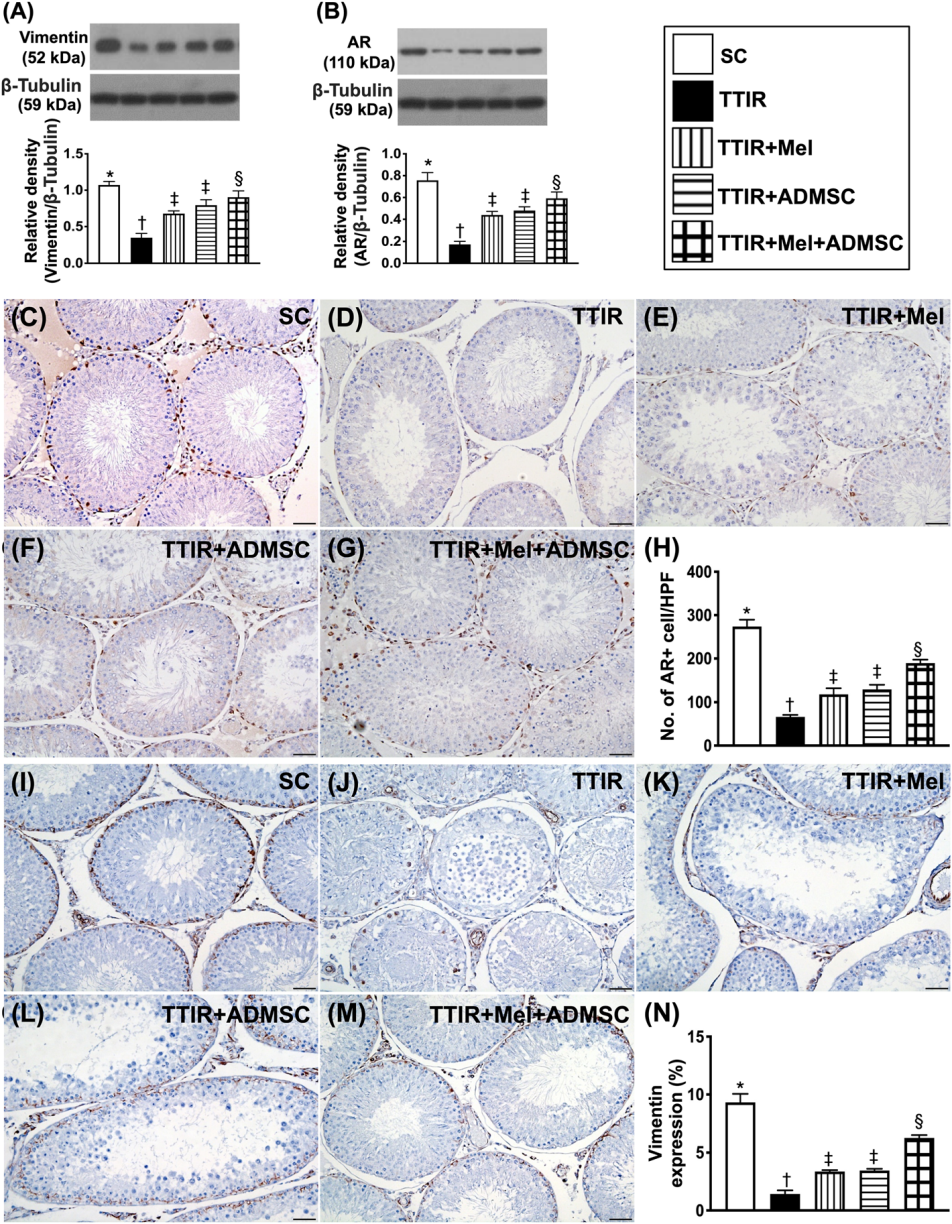
Mel-ADMSCs therapy upregulated the protein and cellular expressions of androgen receptor and vimentin by 72 h after TTIR procedure. **A** Protein expression of vimentin, * vs. other groups with different symbols (†, ‡, §), p < 0.0001. **B** Protein expression of androgen receptor (AR), * vs. other groups with different symbols (†, ‡, §), p < 0.0001. **C**–**G** Illustrating the immunohistochemical (IHC) stain for identification of AR+ cells (gray color). **H** Analytical result of number of AR-positively stained cells, * vs. other groups with different symbols (†, ‡, §), p < 0.0001. **I**–**M** Showing the IHC stain for identification of vimentin + cells (gray color). **N** Analytical result of number of vimentin-positively stained cells, * vs. other groups with different symbols (†, ‡, §), p < 0.0001. All statistical analyses were performed by one-way ANOVA, followed by Bonferroni multiple comparison post hoc test (n = 6 for each group). Symbols (*, †, ‡, §) indicate significance (at 0.05 level)

### Scoring for identification of loss of seminiferous tubule (ST) epithelium and germ cell degradation by 72 h after IR procedure (Fig. 4)

To assess the therapeutic impact of Mel-ADMCSs on protecting the ST, the microscopic examination was performed. The result of H&E stain demonstrated that the thickness of ST, an indicator of intactness of the epithelium, was highest in group 1, lowest in group 2, and significantly higher in group 5 than in groups 3 and 4, but it was similar between groups 3 and 4.

**Fig. 4:**
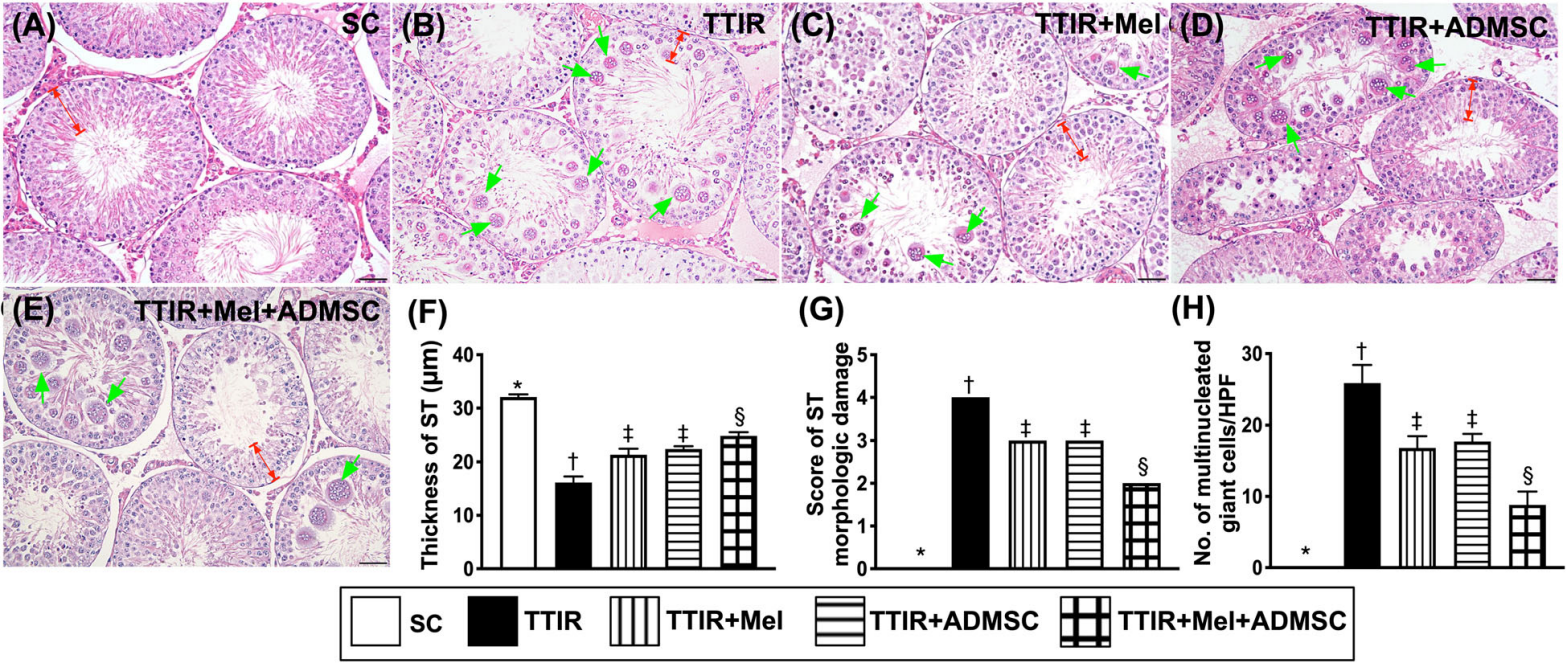
Using histopathological analysis and scoring for identification of seminiferous tubule damage and expression of multinucleated giant cells by 72 h after IR procedure. **A**–**E** Illustrating the microscopic (× 200) of H&E stain for identification of histological feature (i.e., cross-section) of testis. The architectural feature of ST (red color) was remarkably destructed in TTIR groups as compared with the other groups. **F** The analytical result of the thickness of ST (red color of double arrows), * vs. other groups with different symbols (†, ‡, §), p < 0.0001. **G** Score of the severity of ST morphologic damage, * vs. other groups with different symbols (†, ‡, §), p < 0.0001. **H** Some multinucleated giant cells (green arrows) were clearly notably in IR animals than in sham-control animals; analytical result of number of multinucleated giant cells (i.e., germ cell degradation), * vs. other groups with different symbols (†, ‡, §), p < 0.0001. All statistical analyses were performed by one-way ANOVA, followed by Bonferroni multiple comparison post hoc test (n = 6 for each group). Symbols (*, †, ‡, §) indicate significance (at 0.05 level)

On the other hand, the morphologic destruction score of ST, an indicator of ST damage, exhibited an opposite pattern to the thickness of ST among the groups.

It is well recognized that the *germ cells* give rise to the gametes of an organism that reproduces sexually. In histopathological study, we found that the number of the multinucleated giant cells in the lumen of seminiferous tubules, an indicator of germ cell degradation, was highest in group 2, lowest in group 1, and significantly lower in group 5 than in groups 3 and 4 but it showed no difference in groups 3 and 4, suggesting that Mel or ADMSCs treatment was significantly and combined Mel-ADMSCs treatment was more significantly ameliorated TTIR-induced germ cell damage.

### The cellular expression of Sertoli cells and Leydig cells in testis by 72 h after IR procedure (Fig. 5)

Next, we investigated the protective effect of Mel-ADMCSs on the integrity of the Sertoli cells, a part of a seminiferous tubule participating in the process of spermatogenesis and the production of sperm. The microscopic finding of H&E stain demonstrated that the number of Sertoli cells was highest in group 1, lowest in group 2, and significantly higher in group 5 than in groups 3 and 4, but it was similar between groups 3 and 4.

**Fig. 5:**
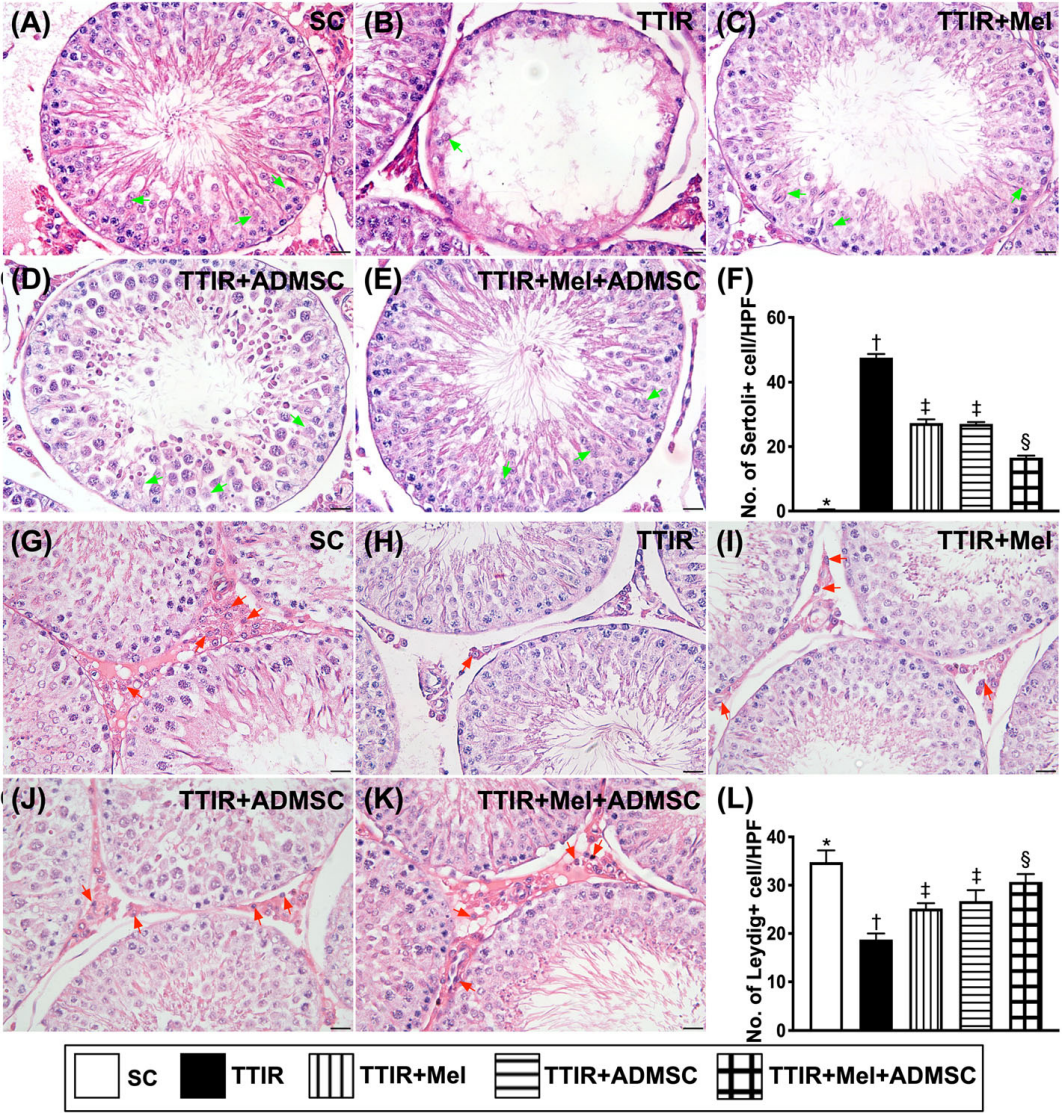
Mel-ADMSCs therapy effectively protected Sertoli cells and Leydig cells against TTIR injury by 72 h after IR procedure. **A**–**E** Illustrating the microscopic (× 400) finding of H&E stain for identification of histological feature (i.e., cross-section) of Sertoli cells (green arrows). The result showed that number of Sertoli cells was notably reduced in TTIR groups than in other groups. **F** The analytical result of the number of Sertoli cells, * vs. other groups with different symbols (†, ‡, §), p < 0.0001. **G**–**K** Illustrating the microscopic (× 400) finding of H&E stain for identification of histological feature (i.e., cross-section) of Leydig cells (red arrows). The result demonstrated that number of Leydig cells was notably reduced in TTIR groups than in other groups. **L** The analytical result of the number of Leydig cells, * vs. other groups with different symbols (†, ‡, §), p < 0.0001. HPF, high-power field. All statistical analyses were performed by one-way ANOVA, followed by Bonferroni multiple comparison post hoc test (n = 6 for each group). Symbols (*, †, ‡, §) indicate significance (at 0.05 level)

Additionally, Leydig cells are well known to be interstitial cells located adjacent to the seminiferous tubules in the testes and their function is to produce the androgen and testosterone. In this way, we also investigated the protective effect of Mel-ADMCSs on the integrity of the Leydig cells. As we expected, the H&E stain demonstrated that the number of Leydig cells exhibited a similar pattern of Sertoli cells among the groups.

### The cellular expression of alpha-microtubule and CD68 in testis by 72 h after IR procedure (Fig. 6)

It is well known that alpha-microtubule (α-MT)-based cytoskeleton is crucial to confer spermatid and organelle transport. The result of the present study demonstrated that the number of α-MT was highest in group 1, lowest in group 2, and significantly higher in group 5 than in groups 3 and 4, but it was similar between groups 3 and 4, suggesting that combined Mel-ADMCSs was superior to either one alone for protecting this testicular apparatus against IR damage. On the other hand, the cellular expression of CD68, an indicator of cellular level of inflammation, displayed an opposite pattern of α-MT among the groups, implicating that Mel-ADMCSs was superior to either one alone for protecting the testis against inflammatory damage.

**Fig. 6:**
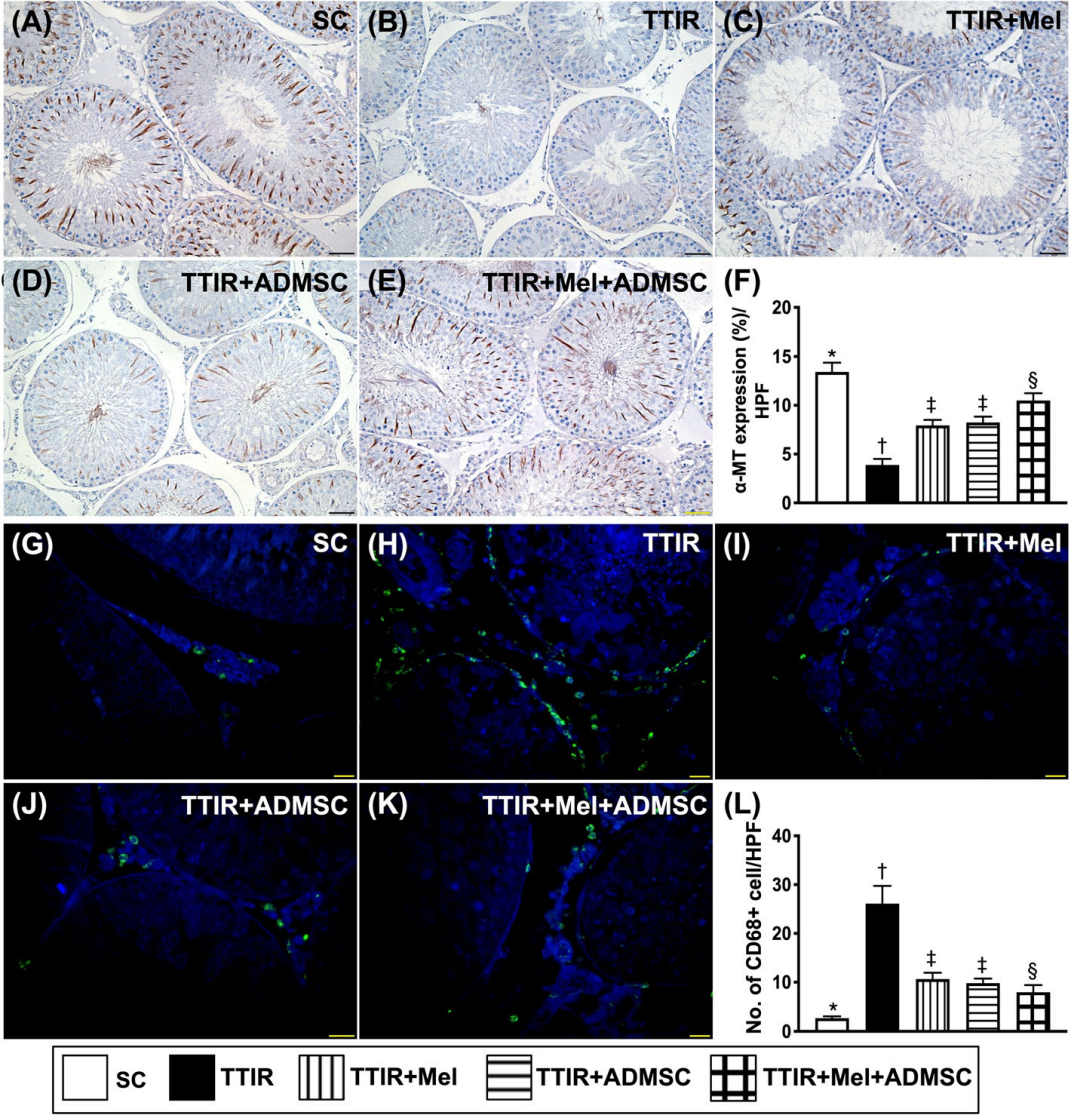
Mel-ADMSCs therapy preserved the integrity of alpha-microtubule and suppression of cellular expression of CD68 in testis by 72 h after IR procedure. **A**–**E** Illustrating the microscopic finding (× 400) of immunohistochemical stain for identification of the expression of alpha-microtubule (α-MT) (spindle shape, gray color). **F** Analytical result of number of α-MT, * vs. other groups with different symbols (†, ‡, §), p < 0.0001. **G**–**K** Illustrating the immunofluorescent (IF) microscopic finding (× 400) for identification of CD68-positively stained cells (green color). **L** Analytical result of number of CD68+ cells, * vs. other groups with different symbols (†, ‡, §), p < 0.0001. HPF, high-power field. All statistical analyses were performed by one-way ANOVA, followed by Bonferroni multiple comparison post hoc test (n = 6 for each group). Symbols (*, †, ‡, §) indicate significance (at 0.05 level)

### The cellular level of inflammatory reaction in testis by 72 h after IR procedure (Fig. 7)

Undoubtedly, inflammatory reaction plays a key role on tissue/organ damage after ischemic or IR attack. In the present study, IHC stain was utilized for delineating the impact of Mel-ADMCSs on restraining the IR from damaging the testis. As we expected, the cellular expression of MMP-9 and MPO, two indicators of inflammation, were lowest in group 1, highest in group 2, and significantly lower in group 5 than in groups 3 and 4, but they did not differ between groups 3 and 4.

**Fig. 7:**
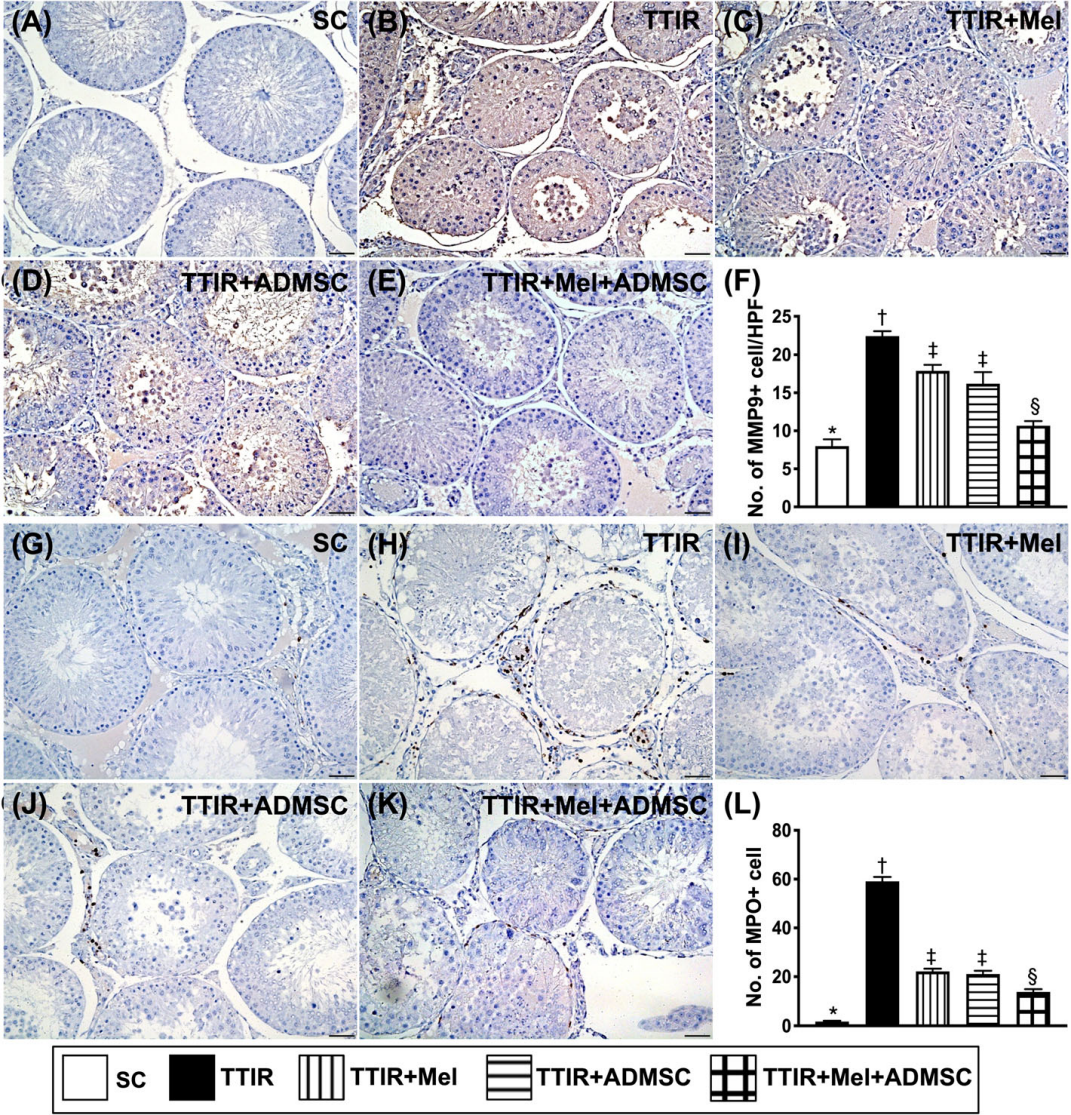
Mel-ADMSCs therapy effectively inhibited the cellular level of inflammatory reaction in testis by 72 h after IR procedure. **A**–**E** Illustrating the microscopic finding (× 400) of immunohistochemical (IHC) stain for identification of positively stained matrix metalloproteinase (MMP-9) cells (gray color). **F** Analytical result of number of MMP-9+ cells, * vs. other groups with different symbols (†, ‡, §), p < 0.0001. **G**–**K** Illustrating the microscopic finding (× 400) of IHC stain for identification of positively stained myeloperoxidase (MPO) cells (gray color). **L** Analytical result of number of MPO+ cells, * vs. other groups with different symbols (†, ‡, §), p < 0.0001. All statistical analyses were performed by one-way ANOVA, followed by Bonferroni multiple comparison post hoc test (n = 6 for each group). Symbols (*, †, ‡, §) indicate significance (at 0.05 level)

## Discussion

This study which investigated the therapeutic impact of Mel-ADMCSs on protecting the testis against TTIR injury yielded several striking preclinical implications. First, we successfully created TTIR animal model for answering key questions in the current study. Second, the result of the present study demonstrated that Mel was comparable to ADMSCs for attenuating the IR-induced testicular injury. Third, the result of the present study further showed that combined Mel and ADMCSs was superior to either one alone on protecting the testis against the TTIR injury, highlighting that this therapeutic management may be potential for the TTIR injured patients, especially for those who are refractory to the conventional therapy for preserving fertility.

Abundant clinical studies have identified that testicular torsion is a serious urological emergency that is most frequently encountered in the newborns, children, and adolescent males [1, 2]. Despite utmost importance in rapid diagnosis and prompt treatment [3–5], majority of the patients still have profound damage due to the fact that testicular is an intrinsically vulnerable organ, resulting in the potentially grave sequelae of infertility and subfertility. It is well recognized that reperfusion injury to the organ is always persistent for few hours to several days, raising a critical issue to be paid attention in all IR organs. In this way, it is believed that even undergoing early and successful surgical intervention in acute TTIR patients, reperfusion-induced testicular damage is still present. This suggests that the conventional interventions are indeed lacking much to be desired, raising the need of considering a new and safe supplementary treatment to ameliorate the development of grave sequelae of infertility and subfertility in acute TTIR patients just after the surgical procedure.

An association between organ IR injury and generation of oxidative stress and vigorous inflammatory reaction has been extensively investigated [24, 29–31, 33]. Studies have further demonstrated that Mel-ADMSCs therapy effectively protected the IR tissue/organs mainly through ameliorating the molecular-cellular perturbations [29–31]. An essential finding in the present study was that the inflammatory and oxidative stress biomarkers were markedly increased in TTIR animals than in those of SC group. In this way, our findings, in addition to being consistent with the findings of the previous studies [24, 29–31, 33], could explain the testicular macro- and micro-structural damage in this TTIR animals. However, our results further demonstrated that Mel and ADMSCs were comparable and combined Mel-ADMSCs therapy was superior to either one on suppressing these molecular-cellular perturbations and protecting the integrity of testicular architectures. Accordingly, our findings corroborated the findings of these previous reports [29–31].

A link between the higher the protein expressions of apoptotic, fibrotic, and mitochondrial-damaged biomarkers in IR organs, the more severe the damage of the organs [24, 26, 29–31], implicating that these are useful prognostic biomarkers in setting of IR injury. A principal finding in the present study was that the protein expressions of apoptosis, fibrosis, and cytosolic cytochrome C (i.e., a mitochondrial damaged marker) were substantially increased in TTIR group than in the SC group. Our findings, in addition to being consistent with the findings of those previous studies [24, 26, 29–31], could at least in part explained why the testicular macro- and micro-structural were deeply destructed in TTIR animals. Importantly, these parameters were notably improved by either Mel or ADMSCs treatment and further remarkably reversed by combined Mel-ADMSCs treatment.

The most important finding in the present study was that numbers of Sertoli cells, AR cells, seminiferous tubule epithelium (i.e., the architectures of testis), and the vimentin (i.e., ultrastructure of the cells) as well as the thickness of epithelium were substantially reduced, whereas the number of germ-cell degradation was remarkably increased in the TTIR group than in the SC group. However, these testicular damaged parameters were notably alleviated by Mel or ADMSCs therapy and further significantly reversed by combined Mel-ADMSCs therapy, highlighting that the Mel-ADMSCs treatment should be earnestly considered as a potential option for those patients who suffer from disastrously TTIR injury.

### Study limitation

This study has limitations. First, although the results of the present study were attractive and promising, the study period was relatively short (i.e., the study period was only 72 h). Thus, the long-term impact of Mel-ADMSCs therapy on protecting the testis against TTIR damage remains uncertain. Second, despite the molecular-cellular level of AR was significantly preserved in TTIR animals undergoing Mel-ADMSCs, the functional integrity of these TTIR animals was not assessed. Accordingly, the fertility ability (defined as the final success of therapy by Mel-ADMSCs) of these animals is still currently unclear. Third, in the present study, randomly choosing just three fields from each slide rather than choosing systematic random sampling would have bias.

In conclusion, the result of the present study demonstrated that combined Mel-ADMSCs therapy offered additional benefit on protecting the testis against TTIR injury.

## Data Availability

The data that support the findings of this study are available from the corresponding authors upon reasonable request.

## Abbreviations

α-MT: Alpha-microtubule
ADMSCs: Adipose-derived mesenchymal stem cells
AR: Androgen receptor
DNA: Deoxyribonucleic acid
ECL: Enhanced chemiluminescence
H&E stain: Hematoxylin and eosin stain
HPFs: High-power fields
IF: Immunofluorescent
IHC: Immunohistochemistry
IR: Ischemia-reperfusion injury
Mel: Melatonin
MMP: Matrix metalloproteinase
MPO: Myeloperoxidase
NOX: NADPH oxidase
PARP: *Poly (ADP-ribose) polymerase*
PVDF: Polyvinylidene difluoride
ROS: Reactive oxygen species
SC: Sham-operated control
SDS-PAGE: Sodium dodecyl sulfate-polyacrylamide gel electrophoresis
ST: Seminiferous tubules
TBS: Tris-buffered saline
TGF-ß: Transforming growth factor Beta
TTIR: Testicular torsion-induced ischemia-reperfusion injury
